# Human Antimicrobial Peptides as Therapeutics for Viral Infections

**DOI:** 10.3390/v11080704

**Published:** 2019-08-01

**Authors:** Aslaa Ahmed, Gavriella Siman-Tov, Grant Hall, Nishank Bhalla, Aarthi Narayanan

**Affiliations:** 1 National Center for Biodefense and Infectious Disease, School of Systems Biology, George Mason University, Manassas, VA 20110, USA; 2 United States Military Academy, West Point, NY 10996, USA

**Keywords:** human antimicrobial peptides, antiviral strategies, defensins, cathelicidins, hepcidins, transferrins

## Abstract

Successful in vivo infection following pathogen entry requires the evasion and subversion of multiple immunological barriers. Antimicrobial peptides (AMPs) are one of the first immune pathways upregulated during infection by multiple pathogens, in multiple organs in vivo. In humans, there are many classes of AMPs exhibiting broad antimicrobial activities, with defensins and the human cathelicidin LL-37 being the best studied examples. Whereas historically the efficacy and therapeutic potential of AMPs against bacterial infection has been the primary focus of research, recent studies have begun to elucidate the antiviral properties of AMPs as well as their role in regulation of inflammation and chemoattraction. AMPs as therapeutic tools seem especially promising against emerging infectious viral pathogens for which no approved vaccines or treatments are currently available, such as dengue virus (DENV) and Zika virus (ZIKV). In this review, we summarize recent studies elucidating the efficacy and diverse mechanisms of action of various classes of AMPs against multiple viral pathogens, as well as the potential use of human AMPs in novel antiviral therapeutic strategies.

## 1. Introduction

Found in virtually all organisms, antimicrobial peptides (AMPs) are short, positively-charged oligopeptides that exhibit a diversity of structures and functions. AMPs are a fundamental component of the innate immune system and play a vital role in the initial immune response generated against both injury and infections. AMP-mediated immune responses are rapidly activated following infections as AMPs are primarily synthesized and stored in cells of myeloid origin and epithelial cells, among the first responders to infections. AMPs are expressed in a wide variety of tissues including skin, eyes, oral cavity, ears, airway, lung, female reproductive tract, cervical-vaginal fluid, intestines, and urinary tract [1,2]. The majority of AMPs are synthesized as large polyprotein precursors, the proteolytic processing of which releases active peptide segments which can be present alone or in multiple copies. Removal of signal peptide may be a post-translation or a co-translational process. Processed functional peptides have been characterized into many classes in mammals; in humans, they include defensins, cathelicidins, transferrins, hepcidin, human antimicrobial proteins, dermcidin, histones, AMPs derived from known proteins, chemokines, and AMPs from immune cells, antimicrobial neuropeptides, and Beta-amyloid peptides [3]. While all of the AMPs classes have been shown to possess antimicrobial activity, only a few classes have demonstrated antiviral properties.

A defining feature of AMPs is their rapid response to infections of bacteria, viruses, fungi, or protozoa [1,4]. Exhibiting inhibitory and immunomodulatory properties, AMPs have been intensively studied as alternatives to antibiotics in bacterial infections and in recent years have gained substantial attention as viral therapeutics [5]. Here we report on the application of human AMPs in the treatment of viral infections.

## 2. Defensins

### 2.1. Expression

Highly abundant and widely distributed, defensins modulate immune responses thereby playing a central role in innate immunity [6,7,8]. Defensins are classified into three subgroups: α, β and θ. Although humans do not produce functional members of the θ-defensin family of AMPs, expression of θ-defensin mRNA has been observed in humans. The θ-defensin mRNA contains a pre-mature stop codon which prevents translation; however, functional θ-defensins are present in non-human primates [9]. To date, six α-defensin and 31 β-defensin peptides have been identified in various species [9]. Originally isolated from neutrophils, four of the six distinct α-defensins are termed human neutrophil peptides (HNP-1 through 4). They are also produced by myeloid-lineage cells such as macrophages, natural killer (NK) cells and some classes of T and B-cells. α-defensins 5 (HD5) and 6 (HD6) are expressed in epithelial cells in the small intestine [6,8,9,10]. The β-defensin family of AMPs is commonly expressed in birds and mammals. In humans, three β-defensins (HBD-1 through 3) have been fully characterized and a fourth, HBD-4, was recently identified. β-defensins are primarily expressed by epithelial cells and keratinocytes, but can also be produced by neutrophils, macrophages, mast cells, NK cells, dendritic cells, and lymphocytes [6,7,8,10]. Current data suggest a functional redundancy when comparing the efficacy of α and β defensins against various pathogens [11].

Defensins are defined by the presence of a conserved spacing pattern comprised of cysteine residues, which is critical for the efficacy of their cationic antimicrobial properties [9,12]. Human α-defensins are composed of 29 to 34 amino acids with an overall positive charge [9,12,13]. Defensins exhibit a characteristic β-sheet structure with a distinctive six-cysteine motif for which stabilization is a consequence of the presence of three intramolecular disulfide bonds. The α-defensins are synthesized as pre-propeptides consisting of a N-terminal signal sequence, an anionic pro-peptide, and a C-terminal mature peptide comprised of approximately 30 amino acids. HNP1, HNP2, and HNP3 are synthesized by promyelocytes and stored in primary neutrophil granules as mature peptides [10]. In contrast, β-defensins have a short N-terminal pro-region and can retain antimicrobial activity in full-length form, and; therefore, do not require N-terminal processing to be fully active [14]. They are synthesized in epithelial compartments and can range from 38 to 42 amino acids in length.

### 2.2. Antiviral Activity of Defensins

The antiviral activity of defensins was first reported in 1986 [10]. Since then, defensins have demonstrated protection against human immunodeficiency virus (HIV), influenza A virus (IAV), human adenovirus (HAdV), severe acute respiratory syndrome coronavirus (SARSC), papillomavirus (HPV), respiratory syncytial virus (RSV), and herpes simplex virus (HSV) [5,10,15,16,17,18]. Recent studies have focused on elucidating the multiple mechanisms associated with defensins’ antiviral activity (Table 1). Defensins can block viral infection through direct action on virus particles or interfere indirectly at various stages of the viral life cycle [10,18]. Available data suggest antiviral activity occurs predominantly at viral entry steps; however, antiviral effects at other stages of infection have also been reported, particularly affecting viral trafficking within infected cells [19]. Defensins can also modify the innate immune response to viral infections, including: modulation of T-cells, macrophage and dendritic cells recruitment to sites of infection, wound healing and angiogenesis, differentiation and maturation of dendritic cells, induction of the production of pro-inflammatory cytokines by macrophages, mast cells, and keratinocytes, and regulation of cell death pathways [9]. For example, HBD-3 can suppress activation of the caspase cascade to prevent apoptosis in infected cells [20]. Similarly, the concentration of HNPs released into the microenvironment upon activation of neutrophils during inflammation exerts a differential effect on cytokine production in activated monocytes [19]. HNP concentrations of 1 to 10 nM can upregulate the expression of tumor necrosis factor α (TNF-α) and interleukin-1β (IL-1β), whereas concentrations of 10 to 100 µM are cytotoxic to monocytes.

### 2.3. Adenovirus

Human adenovirus (HAdV) is a non-enveloped double-stranded DNA virus that is capable of infecting the respiratory, gastrointestinal, ocular, and excretory systems in humans. There are approximately 80 recognized HAdV serotypes, subdivided into species A–G [21,22]. Currently there are only a limited number of HAdV therapeutic strategies and vaccines available to treat HAdV infections. Alpha defensins have demonstrated an ability to hinder HAdV infections in vitro [21,23]. HD5 reduces HAdV replication by 95% when cells are exposed to the peptide (IC_50_ = 3–4 µM) prior to infection, and by 50% when peptide is added 30–60 min post inoculation, suggesting that inhibition occurs at an early stage during viral infection [21]. Additional studies have shown that direct binding of HD5 (10 µM) to HAdV particles prior to infection prevents the release of internalized viral particles from endosomes [24]. Subsequently, viral particles appear to colocalize with lysosomes indicating altered viral trafficking following infection as a consequence of HD5 binding [24]. These findings suggest defensins’ antiviral activity against HAdV results in blockage of HAdV uncoating and genome exposure [24]. In addition, HD5 antiviral activity is species specific; pre-treatment with 15µM HD5 decreased HAdV infectivity of subspecies A–C and E, while infectivity of HAdV subspecies D and F demonstrated no change [23]. The cause of species specificity of HD5 activity is yet to be determined.

### 2.4. Influenza A Virus

During the early infiltrate in influenza A virus (IAV), neutrophils predominate in infected airways, highlighting their importance in initiating immune responses against IAV [13]. Defensins are; thus, likely to interact with IAV. Neutrophil extracellular traps (NETs) displaying HNPs are formed in vivo and in vitro in response to IAV infection [13]. Cells incubated with defensins pre- or post-infection demonstrated minimal inhibitory activity against IAV, whereas incubation of HNPs with virions prior to infection is necessary for the antiviral activity of these AMPs against IAV (e.g., HNP1 IC_50_ < 2 µg/mL [25]) [13,26]. In addition, the binding activity of defensins against IAV is increased by formation of multi-molecular assemblies of defensins, which may be responsible for pore formation in the IAV envelope, thereby destabilizing virions prior to receptor binding and cellular entry [13].

Expression of β-defensins HBD1, HBD2, and HBD3 has been reported in various epithelial cell tissues, with each β-defensin demonstrating a unique expression induction profile in response to IAV infection [13]. However, HBD1 and HBD2 have also been detected in monocytes, macrophages and monocyte-derived dendritic cells (DCs), and possess strong neutralizing activity against multiple IAV strains [10,13]. HBDs exhibit low potency as direct inhibitors of IAV virions, but are speculated to play important immunomodulatory roles by limiting inflammation during IAV infection [13]. While the exact sequence of immunomodulatory events is yet to be determined, it has been reported that deletion of the HBD1 analog in mice resulted in a more serious inflammatory reaction to IAV [13,27]. In addition, HBD3 has demonstrated strong anti-inflammatory effects in cells stimulated with 50 ng/mL lipopolysaccharide (LPS), confirmed by the inhibition of expression of inflammatory mediators such as (TNF-α) [28]. Conversely, α-defensins inhibit IAV replication in infected cells. Pre-incubation of virions with HNP-1 (25 µg/mL) is capable of reducing the replication of IAV strain H1N1 by 10- to 1000-fold in multiple cell lines when compared to replication in untreated cells [29]. Similarly, HNP-1 and 2 can reduce infectious virus of the Phil82 strain of IAV by 85% to 90% in various cell lines [26].

### 2.5. Human Immunodeficiency Virus

Defensins demonstrate antiviral activity against human immunodeficiency virus (HIV), mediated by direct virus–peptide interaction and/or inhibition of viral genome replication. Inhibition mediated by the direct binding of defensins with HIV virions is attributed to interactions between positively-charged HNPs and negatively-charged moieties of the HIV envelope glycoprotein gp120. HNP1, HNP2, and HNP3 function as lectins by directly blocking the interaction of gp120 and the HIV receptor CD4. However, the exact mechanism of this interaction is not well characterized [10,13]. HNP-1 can also interfere with critical steps in the HIV replication cycle [30]. HNP-1 inhibits protein kinase C signaling, which is important for the transcription and nuclear import of the HIV genome [31]. HD5 exhibits a robust dose-dependent (IC_50_ = 400 nM) suppression of HIV-1 replication in absence of serum when pre-incubated with virions [32]. HD5 also blocks HIV-1 infection at a step prior to viral entry [32]. HD5 competitively binds to the CD4 receptor in a dose-dependent manner against HIV, thereby blocking HIV entry into target cells [32]. Interestingly, in contrast to HNP1, HNP2, HNP3, and HD5, HNP4 does not interact with CD4 or HIV gp120 [13]. HNP4 inhibits HIV replication with greater effectiveness than HNP1, HNP2, and HNP3, but it is unclear whether efficacy of HNP4 is mediated only through a direct effect on virions or also on host processes that ultimately affect viral replication.

β-defensins can also exert antiviral activities against HIV. Expression of HBD2 and HBD3 can be induced by microbial products such as endotoxins, viruses, bacteria, and pro-inflammatory cytokines such as TNF and IL-1β [13]. Expression of HBD2 and HBD3, but not HBD1, mRNA can be induced by HIV in human oral epithelial cells. HBD2 inhibits the formation of early HIV transcript products but does not affect cell–cell fusion [10].

### 2.6. Herpes Simplex Virus

Alpha and β-defensins can exhibit anti-HSV properties [33]. HNP-1-4 and HD6 inhibit HSV binding to its target receptor by directly interacting with either the HSV glycoprotein or by binding to heparan sulfate (HS), thereby preventing viral entry [8,33]. HBD3 binds either the HSV receptor or the HSV glycoprotein, thereby eliciting a stronger inhibition of viral entry. Furthermore, treatment of infected cells with defensins post-infection results in substantial reduction in viral replication, indicating that these peptides can exhibit post entry antiviral effects [33]. In addition, studies exploring HNP-1-3 have demonstrated their ability to reduce intracellular HSV protein transport and expression during infection [34]. HNP-1 (100 µg/mL) exhibits the greatest antiviral potential against HSV, reducing HSV titers up to 100,000-fold upon a combination of pre- and post-treatment of infected cells as compared to HNP-2 and HNP-3 (100 µg/mL), which can reduce titers by up to 100-fold following treatment [33].

### 2.7. Respiratory Syncytial Virus

The antiviral effects of human defensins against RSV are relatively unexplored. A study assessing leukotriene B4 (LTB4) stimulation of nasal neutrophil activity highlighted α-defensins as a possible source of antiviral activity against RSV [35]. Cells pre-treated with HBD-2 (4 µg/mL), can reduce RSV viral titers 100-fold following infection [36]. Electron microscopy images revealed damage to the lipid envelope of RSV following HBD-2 treatment, suggesting that defensins destabilize RSV virion envelopes, thereby inhibiting viral cellular entry [36]. The use of defensins in anti-RSV therapies may also limit viral evolutionary strategies that counters antiviral activities, due to the difficulty of changing the viral envelope lipid composition [37]. The evolutionary longevity of defensins suggests this to be a favorable strategy, making defensins an attractive therapeutic candidate for the treatment of RSV infections [16].

### 2.8. Human Papilloma Virus

Most α-defensins possess some level of anti-human papilloma virus (HPV) activity, with HD-6 being a notable exception [38]. Due to their low toxicity and high efficacy, HNP-1 and HD-5 have been most frequently tested as anti-HPV candidates. Recent studies have focused solely on HD5 as it is secreted by epithelial cells in the genitourinary tract. The antiviral activity of HNP-1 and HD5 (5 µg/mL) against HPV is time-independent, with robust inhibition even when peptides are introduced to cells six hours post infection in vitro [38]. Employing immunofluorescent confocal microscopy, the authors demonstrated that the peptides do not inhibit viral entry but rather prevent virion escape from cytoplasmic vesicles [38]. During the course of HPV entry, cleavage of HPV L2 capsid protein by furin, a cellular protease, is required for successful infection [39]. HD5 directly disrupts this proteolytic processing step, thereby preventing HPV genome escape from endosomes [39]. Interestingly, the use of a furin-cleaved HPV does not result in abrogation of HD5 activity. HD5 can still block HPV infection by preventing viral capsid dissociation from the genome, and by reducing viral trafficking within the host cells [40]. These results indicate that α-defensins, particularly HD5, demonstrate robust anti-HPV activity by targeting multiple steps during viral life cycle.

## 3. Cathelicidin, LL-37

### 3.1. Expression

Cathelicidins are peptides with a conserved 100-amino-acid cathelin domain, a protein sequence first identified in porcine leukocytes that is capable of inhibiting the protease cathepsin-L [9]. Cathelicidins are typically linear peptides that fold into amphipathic α-helical structures which are frequently cleaved from the highly variable C-terminal antimicrobial domain [12]. In humans, cathelicidin is produced by a vitamin D-dependent antimicrobial pathway [41]. Although there are multiple cathelicidins found in nature, humans only express a single cathelicidin, known as human cationic antimicrobial peptide 18 (hCAMP-18), hCAP18, or LL-37. LL-37 was identified and isolated in 1995 from neutrophils [1,42]. Like α-defensins, cathelicidins are synthesized as pre-propeptides; following proteolytic removal of the signal peptide, the inactive propeptide is tagged for storage in neutrophil granules. The active cationic molecule is generated by cleavage of the C-terminus end of the hCAP18 precursor protein yielding a linear 37-amino-acid-long peptide [1]. The name hCAP18 alludes to the molecular weight of the polypeptide (18 kDa) and the cationic character of the structure, whereas LL-37 refers to the 37 amino acid length of the peptide along with a Leu-Leu motif located at the N-terminus [1]. The peptide can also be produced in epithelial cells and may play an important role in the initial immune response to various pathogens [10,11]. In epithelial skin cells, LL-37 is further cleaved into shorter segments exhibiting potent antimicrobial activity [43]. LL-37 is also produced by monocytes, NK cells, mast cells, B cells, colon enterocytes, and keratinocytes [1], and has been detected in numerous tissues and biological fluids such as sweat, breast milk, wound fluid, vernix, tracheal aspirates of newborns, and seminal plasma [9]. The concentration of LL-37 and its precursor in tissues and body fluids can range between 2 and 5 μg/mL (0.4–1 μM); however, concentrations can increase up to 20 μg/mL (2.2 μM) during infections in bronchoalveolar fluid [44]. In nasal secretions LL-37 concentrations can vary from 1.2–80 μg/mL [44]. The expression of LL-37 is regulated by a number of endogenous factors including pro-inflammatory cytokines and growth factors such as the active form of vitamin D [1]. LL-37 functions as a chemoattractant for neutrophils, monocytes, dendritic and T-cells and is rapidly released by epithelial cells and leukocytes following infection [11,45]. LL-37 can stimulate IL-6 production in human dendritic cells and may act as both an anti and pro-inflammatory factor during the immune response to various infections [45]. Individuals with cathelicidin deficient neutrophils display an increased susceptibility to infection [11].

### 3.2. Antiviral Activity

The antiviral activity of LL-37 has been reported against a number of viruses including HIV-1, IAV, RSV, rhinovirus (HRV), vaccinia virus (VACV), HSV, ZIKV, and hepatitis C virus (HCV), mediated primarily by its interaction with the virus outer envelope (Table 1) [18,41,43,46]. LL-37 is proposed to remove the outer membrane of viruses in a single event during an antimicrobial attack rather than a gradual piece-by-piece removal [37]. This suggests a carpet model of antimicrobial peptide action, wherein a susceptible membrane remains intact until a threshold concentration of peptide is reached, following which a rapid disintegration of the targeted membrane occurs [47,48].

### 3.3. Influenza A virus

LL-37 therapeutic activity against influenza type A virus has been demonstrated in vivo and in vitro. It is likely that in vivo, IAV encounters LL-37 in the respiratory tract following innate immune responses against the virus and is secreted from neutrophils, macrophages, and epithelial cells [44,49]. Early studies assessed the antiviral activity of LL-37 in vivo using a mouse IAV strain [50]. Mice were nebulized with LL-37 (500 µg/mL) a day prior to infection with a lethal dose of IVA PR/8 mouse strain and survival and weight loss were monitored for 14 days following infection [50]. Initially, all mice exhibited weight loss, but weight loss ceased at day seven in mice treated with LL-37 or the IAV antiviral zanamivir. Mice treated with LL-37 and zanamivir exhibited 60% survival compared to the untreated group which succumbed to infection by day nine suggesting that therapeutic use of LL-37 reduces IAV infection severity in a manner comparable to zanamivir [50]. LL-37 also decreased expression of inflammatory cytokines particularly IL-1β, granulocyte-macrophage colony-stimulating factor (GM-CSF), keratinocytes chemoattractant (KC), and the chemotactic cytokine known as regulated on activation normal t-cell expressed and secreted (RANTES), in bronchoalveolar lavage fluid in mice infected with PR/8 at two days following LL-37 treatment as determined by immunoassay demonstrating the immunomodulatory properties of LL-37 [50]. In vitro plaque assays demonstrated one log inhibition of PR/8 when virus was pre-incubated with LL-37 (50 µg/mL) in Madin-Darby canine kidney (MDCK) cells [50].

During IAV infection, in vitro LL-37 treatment did not prevent viral uptake, cause viral aggregation, and was not associated with blocking of hemagglutinin (HA). Interestingly LL-37 inhibits IAV replication at post-entry steps prior to viral RNA or protein synthesis [44]. A reduction in viral load, direct antiviral effects in epithelial cells, and inflammatory cytokine production have all been linked to LL-37 activity [49]. LL-37 inhibits the NY01 strain of IAV with a significant reduction in uptake of virus into cells, and in a manner dependent on dosage [44,49]. For optimal anti-IAV activity the central helix of LL-37 is required, as evident by fragments of LL-37 containing the complete central sequence of the peptide demonstrating more robust antiviral responses as compared to fragments with shorter central fragments. At a concentration of <2 µM, NY01 was only partially inhibited; however, this inhibition was surprisingly lost at higher concentrations of LL-37 [44]. A strain containing only the pandemic HA (Mex 1:7); however, was inhibited by LL-37 at all concentrations tested (up to 10 µM). Consistent with previous studies, these results suggest the antiviral effects of LL-37 are not determined by direct interaction of LL-37 with the viral HA [44].

Experimental data also demonstrates the participation of LL-37 in host defenses against IAV through modulation of innate immune cells, particularly neutrophils. IAV infection induces a respiratory burst response in neutrophils, and this response is noticeably up-regulated by pre-incubation of LL-37 with IAV [49]. LL-37 alone does not stimulate this respiratory burst response; however, optimal enhancement of this antiviral response is achieved only when the virus is pre-incubated with LL-37 [49]. Furthermore, there is growing evidence that NET formations play an important role during IAV infections [49]. Recently, in vivo studies have provided evidence of NET formation in the lungs of IAV-infected mice [51]. On the other hand, in vitro evidence of binding of IVA to NETs suggests LL-37 induces an increase in NET formation in response to IAV, which may promote viral clearance in vivo [49,51]. Data also suggest that significant protection against IAV may be provided by therapeutic treatment of influenza infected individuals with LL-37 or by increasing natural cathelicidin expression in the IAV-infected lung [11]. Hence, to maximize anti-IAV functions, approaches, such as therapeutic administration of naturally-occurring cathelicidins, as well as increasing vitamin D levels to boost endogenous cathelicidin have been proposed [11].

### 3.4. Human Immunodeficiency Virus

Earlier studies provided evidence of LL-37 ability to protect against HIV-1 infection given epithelial expression of LL-37, including in peripheral blood mononuclear cells such as CD4+ T-cells in vitro [52]. LL-37 directly inhibits the activity of HIV-1 reverse transcriptase via a protein–protein interaction in a dose-dependent manner (IC_50_ = 15 µM) [9,53]. Inhibition of HIV-1 protease activity with LL-37 has also been reported; however, this activity is less potent when compared to inhibition of HIV-1 reverse transcriptase (20%–30% inhibition at 100 µM). In addition, the plasma levels of LL-37 in HIV positive individuals undergoing antiretroviral therapy (ART) are much higher than in patients who are not, corresponding with an increased susceptibility to secondary infections in patients not undergoing ART [54].

### 3.5. Dengue Virus

To date, little research has been performed to characterize the antiviral activity of LL-37 against DENV. However, a recent study demonstrated that treatment of dengue virus 2 (DENV-2) with LL-37 inhibits viral infection in green monkey kidney (Vero) cells. Incubation of virus with LL-37 (10-15 µM) prior to infection inhibits production of viral particles, whereas pre-treatment of cells with LL-37 demonstrates no effect on viral replication [43]. Molecular docking studies of DENV-2 E protein have revealed the direct binding of LL-37 with E2 protein moieties, further demonstrating the peptide’s ability to act as an entry inhibitor [43]. A more recent study assessed truncated and full length variants of LL-37 against other serotypes of DENV which revealed the inhibitory properties of LL-37 required the full length peptide [55]. In comparison to DENV-2, DENV-4 required higher concentrations of LL-37 for inhibition with effect being not as potent as with the former. DENV-1 and DENV-3 inhibition however, was prominent at lower concentrations of LL-37 [55]. A recent study using DENV-2 infected keratinocytes has demonstrated the production of AMPs by infected cells as well as bystander cells [56]. Pre-incubation of cells with LL-37 prior to DENV-2 infection results in a significant decrease in viral titers and replication in infected keratinocytes, whereas HBD2 and HBD3 demonstrate minimal inhibition [56]. Additionally, as vitamin D is an inducer of LL-37 expression, supplementation of populations with vitamin D prior to DENV outbreak seasons has been suggested as a possible preventative strategy to control the virus at initial stages of infection [43].

### 3.6. Respiratory Syncytial Virus

A few studies have demonstrated the efficacy of LL-37 against RSV [11,57]. Cells pre-incubated with LL-37 (>10 µg/mL) are protected against RSV infection whereas addition of LL-37 two hours post-infection results in decreased antiviral activity [57]. Additionally, LL-37 can limit viral-induced cell death in infected cell cultures indicating that the peptide’s activity is not limited to prophylactic treatment. Treatment of epithelial cells with LL-37 prior to infection results in peptide internalization and retention, which provides antiviral protection for several hours post-treatment [57]. Furthermore, RSV infection induces the production of cytokines and chemokines in lungs. LL-37 (50 µg/mL) can impact the expression of chemokines as well as viral load when pre-incubated with RSV [11]. While the exact mechanism of the antiviral activity of LL-37 against RSV is not well established, it is speculated that the peptide directly interacts with the virus prior to infection due to its dose-dependent early effects on RSV infection. Interestingly, children with lower cathelicidin levels are more susceptible to RSV infection and display an increase in the severity of RSV-associated bronchitis [58].

### 3.7. Human Rhinovirus

Human rhinoviruses (HRVs) are causative agents of the common cold and most viral respiratory tract infections. As respiratory epithelial cells are the primary targets of HRV infection, studies evaluating the efficacy of LL-37 on HRV have utilized airway epithelial cells. LL-37 (50 µg/mL) demonstrates direct antiviral activity against HRV when added as a pre-treatment by acting on viral particles, and when added post infection by acting on the host cell [59]. LL-37 can induce a significant reduction in the metabolic activity of infected cells, as measured by mitochondrial metabolic potential [59]. Studies evaluating HRV in cystic fibrosis cells have revealed that expression of LL-37 decreases HRV viral load in vivo [60]. Thus, LL-37 reduces HRV infections in respiratory cells as well as in cystic fibrosis cells.

### 3.8. Vaccinia Virus

Vaccinia virus (VACV) is a DNA virus that can infect many types of mammalian cells. LL-37 limits VACV replication and can alter viral membranes [61]. VACV gene expression and viral titers are reduced in a dose-dependent manner in cells pre-incubated with LL-37(25–50 µM) [61]. Transmission electron microscopy images have shown a disruption in the integrity of VACV viral membrane after 24 h incubation with LL-37. Whereas murine LL-37 has demonstrated great efficacy and protection against VACV during infection, the efficacy of human LL-37 against VACV is unknown [61].

### 3.9. Herpes Simplex Virus

Few studies evaluating the efficacy of LL-37 against HSV-1 have been performed, all of which assessed LL-37 inhibition of HSV-1 in the context of a corneal infection [62]. LL-37 (500 µg/mL) can inhibit HSV-1 infection when pre-incubated with virions in vitro [62]. LL-37 reduces viral titers in corneal epithelial cells by more than 100-fold when compared to a scrambled LL-37 control [62]. Another study evaluating the anti-HSV-1 activity on corneal implants assessed the release of LL-37 delivered through corneal implant-incorporated nanoparticles [63]. Whereas LL-37 did not clear viruses from infected cells, it blocked HSV-1 infection in corneal epithelial cells by preventing viral-cell attachment [63]. These studies reinforce the mechanism of LL-37 antiviral activity as entry inhibition. Interestingly, LL-37 released from HSV-1 infected keratinocytes can also enhance HIV-1 infection [64]. This study measured the susceptibility of Langerhans cells (LC) to HIV-1 and implicates LL-37 in increasing HIV-1 cell receptor counts, resulting in increased HIV-1 infection [64]. While there are numerous studies linking a decrease in HIV-1 infection as a consequence of LL-37 or defensin treatment, the difference in this activity of the AMPs is possibly attributed to different cell targets.

### 3.10. Zika Virus

Zika virus (ZIKV) is a positive-sense, single-stranded RNA virus that can cause fever, headaches, rashes, joint pain, and myalgia in children and adults, and “microcephaly, ventriculomegaly, intracranial calcifications, abnormalities of the corpus callosum, retinal lesions, craniofacial disorder, hearing loss, and dysphagia” in neonates [65]. The emergence of ZIKV is a global concern since it is the first major infectious disease that has been associated with birth defects in over five decades [66]. Currently, no vaccines or treatments are available to prevent ZIKV infection [66]. He et al. [46] conducted a study to determine whether LL-37 and synthetic derivatives can be used to treat ZIKV infection in primary human fetal astrocytes [46]. Whereas LL-37 is toxic to these cells (EC_50_ = 20 M), an LL-37 derivative, GF-17, can be safely used due to its lower toxicity (EC_50_ > 50 µM) [46]. Treatment of primary human fetal astrocytes with 10 µM of GF-17 24 h after ZIKV infection results in a seven-fold decrease in the number of ZIKV plaque forming units [46]. Pre-incubation of ZIKV between 1 and 4 h with GF-17 (10 µM), results in at least a 95% decrease in the number of active zika virions [46]. In addition to the possibility of GF-17 directly interacting with ZIKV virions as a mechanism of antiviral activity, GF-17 increases interferon-α2 (IFN-α2) expression in a dose-dependent manner, which further impacts the ability of ZIKV to infect primary human fetal astrocytes [46]. The study suggests that GF-17 may be a possible option for the prevention and treatment of ZIKV infections [46].

### 3.11. Hepatitis C Virus

Hepatitis C virus (HCV) is a major worldwide health concern with possible severe outcomes including cirrhosis, liver cancer, and even death if an infection is left untreated [67]. Whereas effective antivirals against HCV infections exist, there is an unmet need for novel anti-HCV treatments that can overcome current treatment barriers such as cost and access to healthcare [41,67]. LL-37 has demonstrated anti-HCV properties in cell culture. HCV titers are significantly reduced when HCV is pre-incubated with LL-37 and subsequently used to infect Huh-7 cells [41]. Although different strains of HCV were utilized in this study, the antiviral effects of LL-37 are not associated or dependent on a specific HCV strain. Decrease in viral replication occurs in a dose-dependent manner [41]. Furthermore, of LL-37 primarily acts against HCV extracellularly, consistent with the activity of LL-37 against other enveloped viruses.

### 3.12. Venezuelan Equine Encephalitis Virus

Venezuelan equine encephalitis virus (VEEV) is an alphavirus that has been categorized as biothreat agent due to ease of aerosolization and high retention of infectivity in the aerosol form [68,69]. Currently there are no FDA approved therapeutics to combat VEEV infections. LL-37 has recently demonstrated anti-VEEV activity in vitro [70]. A significant decrease in intracellular VEEV genomic RNA copies was observed upon pre-incubation of LL-37 (10 µg/mL) and VEEV. Microscopy data revealed the extracellular aggregation of VEEV virions, suggesting the mechanism of action of LL-37 against VEEV is through direct interaction with viral particles, thereby inhibiting entry [70]. Pre-treatment of human microglial cells with LL-37 prior to infection also resulted in a significant reduction in VEEV titers, suggesting entry prevention is not the only mechanism of LL-37-mediated inhibition. Indeed, LL-37 increased the expression of type I interferon (IFNβ), possibly inducing an antiviral state [70]. LL-37 could prove as a potent therapeutic candidate against VEEV and possibly other alphaviruses.

## 4. Transferrins

### 4.1. Expression

The most notable AMP exhibiting antiviral activity of the transferrin family of iron-binding proteins is lactoferrin (LF), a multifunctional 80 KDa glycoprotein [71,72]. Originally discovered in bovine milk, LF is highly conserved and can be found in humans, mice, and porcine species. It is expressed in most biological fluids such as exocrine secretions (milk, saliva, fluids of digestive tract, and tears) and in neutrophil granules [72,73]. LF serves as a key component of innate immune defenses and demonstrates antimicrobial activity against a wide range of bacteria and viruses through direct action on pathogen membrane and target host cell moieties as well as through modulation of inflammation (Table 1) [72,74].

LF expression may be induced under hormonal control by epithelial cells in mammary glands or at well-defined stages of cell cycle such as neutrophil differentiation [74,75]. LF structure consists of a polypeptide chain that is characterized by a highly basic and positively-charged N-terminal region [74,76]. The LF chain folds into two globular lobes linked by a three-turn alpha helix, with each lobe containing an iron-binding site [77]. There is a strong interaction between the two lobes when iron is bound, which renders LF resistant to proteolysis in this holo form as compared to an open apo form [77]. In the stomach, acidic pepsin hydrolysis of the N-terminal of LF yields lactoferricin (Lfcin), a 25-amino-acid peptide with multiple hydrophobic, positively-charged residues [74,76]. Lfcin retains the properties of LF and demonstrates potent antiviral activity.

### 4.2. Respiratory Syncytial Virus

Early studies have demonstrated the direct anti-RSV activity of LF in vitro [78,79,80]. In HEp-2 cells, LF (100 µg/mL) decreased the production of IL-8 induced by RSV infection when pre-incubated with virions. This LF-mediated inhibition was dependent on LF–RSV interactions as LF inhibited RSV entry into HEp-2 cells [79]. Pre-incubation of LF (100 µg/mL) with RSV resulted in decreased viral entry. LF was found to inhibit RSV entry by directly binding to the F_1_ subunit of the RSV F protein, which mediates fusion of the virion with the cell membrane [79].

### 4.3. Influenza Virus and Parainfluenza Virus

Human and bovine LF have demonstrated anti-IAV activity. Bovine LF inhibits IAV-mediated programmed cell death and directly binds viral hemagglutinin leading to inhibition of viral hemagglutination [81,82,83,84]. Only a few studies; however, have evaluated the activity of human LF in the context of IAV infection. In Madin-Darby canine kidney cells, human LF (20-80 µg/mL) demonstrated enhanced antiviral activity against avian IAV in a dose-dependent manner [85]. In addition, bovine LF inhibited parainfluenza virus replication in vitro and decreased viral adsorption onto cells, thereby preventing viral entry [86]. Together these results highlight the possible role of LF in preventing influenza and parainfluenza virus infections.

### 4.4. Adenovirus

Lactoferrin-mediated anti-HAdV activity occurs at multiple stages during infection. In one study LF inhibited HAdV replication during different phases of infection; when the peptide was introduced before infection, after viral adsorption, and when the peptide was present throughout the experiment, indicating more than one mechanism of action may be involved [87]. Bovine LF has also demonstrated anti-HAdV properties in a dose-dependent manner similar to human LF [87,88,89]. Bovine LF demonstrated the greatest inhibition when virus was pre-incubated with LF (1 mg/mL), which was validated by electron microscopy imaging the binding of LF to HAdV, thus suggesting direct LF inhibition as a mechanism of action [89], In contrast, human LF in tear fluids was implicated in promoting HAdV binding to epithelial cells, whereby HAdV hijacks human LF and utilizes the protein in order to bind to host cells [90].

### 4.5. Herpes Simplex Viruses 1 and 2

Studies assessing inhibition of HSV-1 have revealed the potency of LF at inhibiting replication of the viruses. LF (0.5–1 mg/mL) inhibited HSV-1 replication in human embryo lung cells and prevented virus adsorption and entry [91]. Marchetti et al. also demonstrated the ability of bovine and human LF to inhibit HSV-1 replication and adsorption in Vero cells independent of iron-binding [92,93]. Furthermore, intracellular trafficking of virions that gained entry was delayed [94]. Similar inhibitory properties were displayed by Lfcin when the peptide was tested against HSV-1 [94]. In addition, cell-to-cell viral spread was inhibited by LF as well as Lfcin [95]. The data presented by these studies demonstrate LF’s antiviral properties against HSV.

### 4.6. Hepatitis C and B Virus

Lactoferrin has demonstrated efficacy against HCV and HBV. Bovine LF can prevent HCV infection in human hepatocytes and in turn was tested as a measure to control HCV viremia in chronic hepatitis C patients [96]. However, LF only reduced HCV RNA in patients with previously low HCV RNA serum concentration. Conversely, a randomized trial of bovine LF in patients with chronic hepatitis C reported that orally-administered LF did not demonstrate significant efficacy against HCV when compared to a placebo control [97]. Combination therapy using the antiviral molecule ribavirin, LF, and interferon therapy, on the other hand, suggested that LF contributed to decreased HCV RNA titers [98]. These contradictory results suggest that while orally-administered LF may not be a promising stand-alone therapeutic, it can enhance the effectiveness of currently used therapeutics. Studies on the interaction of HCV and LF reported the direct interaction and binding of human and bovine LF with the HCV envelope proteins E1 and E2 [99]. A more recent study concluded that LFs of various species directly prevented HCV cell entry by binding to virions. Furthermore, pre-incubation of virions or post-treatment of infected cells with human, bovine, sheep, or camel LF inhibited HCV replication in human hepatoma (HepG2) cells, suggesting that LF may be used prophylactically as well as therapeutically, particularly in combined therapies [100].

Pre-incubation of HBV with LF had no effect on viral replication; however, pre-treatment of cells with human or bovine LF prevented infection [101]. Bovine LF did not interact with HBV but indirectly protected cells from HBV infection [102]. In addition, iron or zin-saturated LF on HBV-infected HepG2 cells inhibited HBV-DNA amplification suggesting its use as a possible candidate for the treatment of HBV infections [103].

### 4.7. HIV

Circulating LF plasma levels in HIV-1-infected patients was significantly decreased compared to non-infected patients [104], suggesting an important role for LF during HIV-1 infection and disease progression. Experiments assessing the role of LF in HIV-1 infection in peripheral blood mononuclear cells revealed LF (IC_50_ = 9.6 µM) as a potent inhibitor of six clinical isolates of HIV-1 in a dose-dependent manner upon treatment of cells prior to infection [105]. Additionally, synergistic effects of human and bovine LF were assessed in combination with zidovudine, which also demonstrated a dose-dependent inhibition [105]. Bovine LF inhibited the HIV-1 entry process by binding to the viral envelope, and HIV-1 variants resistant to LF exhibit mutations in the viral envelope protein [106]. Bovine LF was found to be a more potent inhibitor than human LF in preventing dendritic cell-mediated HIV-1 transmission between cells by directly and strongly binding to DC-SIGN, a receptor molecule on dendritic cells that mediates HIV-1 internalization [106].

In pediatric HIV-1 infections, LF enhanced responses to antiretroviral therapy by decreasing plasma viral load and modulating the immune system [107,108]. LF treatment alone increased CD4+ cell counts, but a more significant increase was observed when LF was combined with antiretroviral therapy [107]. Result suggested that no HIV-1-related symptoms were evident for the duration of the experiments. However, large-scale studies are required in order to further assess LF’s therapeutic potential against HIV.

### 4.8. Hantavirus

Hantavirus is a zoonotic RNA virus that causes severe human disease characterized by widespread and extensive hemorrhaging [109]. In vitro studies have demonstrated that pre-treatment of Vero cells with LF (ED_50_ = 39 µg/mL) significantly reduces Hantavirus foci number [110]. A combination of LF and ribavirin also significantly reduced the number of viral foci where pre-treatment of cells with LF (400 µg/mL), and treatment with ribavirin (100 µg/mL) post infection completely abolished viral foci [109]. In additional synthesis of viral glycoprotein, G2, and nucleocapsid protein (NP) was delayed in LF pretreated cells. In vivo testing revealed that prophylactic LF treatment (160 mg/kg) can significantly improve survival rates following hantavirus infection in suckling mice by up to 70% when compared with non-treated mice [109].

### 4.9. Human Papillomavirus

Human and bovine LF and Lfcin have been demonstrated to inhibit the internalization of HPV-16 particles, as visualized using an HPV virus-like particle (VLP) that fluoresces after cellular internalization [111]. This inhibition occurred in a dose-dependent manner on HaCaT cells [111]. Bovine LF-mediated inhibition was more potent than human LF. However, both LFs inhibited HPV-5 and HPV-16 [112]. Different synthetic derivatives or variants of human and bovine Lfcin demonstrated selective inhibition of HPV, such that a human variant of Lfcin with amino acids 1 to 49 displayed antiviral activity as well as inhibition of attachment of both HPV strains while the bovine variant (17–42) only inhibited HPV-5 infection [112]. These results suggest that different domains of Lfcin contain selective and specific antiviral properties which can be engineered to target specific infections and viral strains.

### 4.10. Rotavirus

Rotavirus is one of the world’s major causes of gastroenteritis in children. As an effort to develop anti-rotavirus strategies, milk proteins including apo-LF (25 µM) and Fe-LF were assessed for inhibitory properties against rotavirus [113]. Results demonstrated apo-LF to be the most potent inhibitor of rotavirus by binding to viral particles and hindering virus attachment as well as preventing rotavirus hemagglutination [113]. A randomized trial; however, concluded that orally-administered LF did not provide protection against rotavirus infection [114]. This serves as an example that the pronounced inhibition in vitro does not necessarily recapitulate the effects that occurs in vivo.

### 4.11. Other Viruses

Lactoferrin antiviral activity against other viruses including poliovirus, alphaviruses, DENV, and Japanese encephalitis virus (JEV) has been studied. In Vero cells, iron-, manganese-, and zinc-saturated LFs have demonstrated anti-poliovirus properties in a dose-dependent manner when LFs were present either during the entire viral life cycle or during viral adsorption [115]. Against alphaviruses, human LF (200 µg/mL) inhibited infection of BHK-21 cells by Sindbis virus (SINV) and Semliki Forest viruses (SFV) adapted to bind heparan sulfate (HS) upon pre-treatment of cells prior to infection [116]. The LF-mediated inhibition was thought to occur by binding of LF to heparan sulfate moieties, thereby preventing virus–receptor interactions [116]. Bovine LF was tested against JEV and DENV, and was capable of inhibiting replication of both viruses. In JEV, similar to alphaviruses, HS-adapted JEV strains were inhibited by bovine LF, while wild-type strains were not [117]. Bovine LF inhibited binding of DENV to HS by directly interacting with HS. Additionally, morbidity of DENV-infected suckling mice was reduced upon administration of a mixture of DENV and LF as compared to DENV-only-infected mice [118].

## 5. Human Antimicrobial Proteins–Eosinophil Proteins

### 5.1. Expression

Eosinophils contain large cytoplasmic granules that play a critical role in innate immune responses. These granules are storage hotspots for major cationic antimicrobial proteins including eosinophil-derived neurotoxin (EDN) and eosinophil cationic protein (ECP). Both EDN and ECP are human antimicrobial proteins that are active ribonucleases and members of the human RNase A superfamily [119,120]. The antimicrobial properties of RNases were mapped to the N-terminal domain that is conserved among ribonucleases [3]. While both proteins contain characteristic RNase A superfamily structures and catalytic residues, they exhibit antimicrobial activity against both bacteria and viruses (Table 1).

### 5.2. Respiratory Syncytial Virus

Eosinophil-derived neurotoxin (EDN), also known as RNase 2, has demonstrated inhibitory activity against RSV in several studies. In one study, RSV infectivity decreased in a dose-dependent manner when eosinophils were introduced to a suspension of RSV [121]. This property was abolished upon addition of a ribonuclease inhibitor suggesting that an RNase was responsible for the antiviral activity. At 50 nM, a plasmid-constructed recombinant human EDN exhibited a 40-fold reduction in RSV infectivity [121]. A decrease in RSV genomic RNA copies as quantified by RT-PCR upon incubation of EDN with virions suggests EDN ribonuclease activity directly targets extracellular viral particles. EDN may contain unique features that help mediate its antiviral activity as ribonuclease A alone has no effect on RSV infectivity.

Additionally, ECP, which is also known as RNase 3, has been detected in infants with acute bronchiolitis following RSV infection [122]. ECP levels were significantly higher in infants who developed persistent wheezing compared to those that do not after a five-year follow-up [122]. Additionally, while ECP was able to reduce RSV infectivity in the same suspension as EDN, ECP is less effective when used to inhibit RSV alone, demonstrating only a six-fold reduction as compared to EDN at a concentration of 50 nM [121]. Hence while ECP exhibits some inhibitory activity against RSV, it is not sufficient in controlling infection as RSV infection itself induces the production of ECP with no evident antiviral effects. Instead, the production of ECP in infants with bronchiolitis can be used as tool in prediction the risk of wheezing cough development in those infants as higher ECP levels correlate with wheezing cough development.

### 5.3. HIV

In earlier studies, EDN did not demonstrate direct activity against HIV; However, it indirectly induces the production of HIV-inhibiting molecules. Soluble factors produced by hosts such as chemokines, interferons, RNases, and alloantigen-stimulated factors (ASF) have been reported to inhibit HIV-1 replication or binding [123]. A test to determine the soluble HIV-1 inhibitory activity utilized supernatants from mixed lymphocytes of healthy individuals that exhibit anti-HIV-1 activity. While the supernatant exhibited anti-HIV activity, this activity was blocked with antibodies specific for EDN by 58% [123]. Antibodies to RNase A had no effect on the anti-HIV-1 activity, thus ruling out its involvement. In addition, an RNase inhibitor significantly blocked the inhibitory activity indicating that EDN or a possible closely-related RNase is responsible for the HIV-1 inhibitory activity [123]. Later studies reported EDN and a recombinant EDN, containing a four amino acid extension of the N-terminus of END, both exhibit anti-HIV-1 activity independent of time of addition, before, during, or 2 h post infection [124].

## 6. AMPs from Immune Cells

### 6.1. Expression

Immune cells have the ability to produce AMPs such as the protease inhibitors elafin and secretory leukocyte protease inhibitor (SLPI) [3]. Studies investigating AMPs produced by gamma-delta T cells (γδ T cells), recorded production of elafin [125]. Elafin is expressed in tissues such as skin, placenta, genital and gastrointestinal tracts, as well as by cells including neutrophils, epithelial cells, macrophages, and keratinocytes and thus can be found at many surfaces and in secretions [126]. Elafin is a 57-amino-acid single-polypeptide chain produced by the proteolytic cleavage of a precursor molecule called trappin-2 [127,128]. Similarly, SLPI is expressed by neutrophils, macrophages, epithelial cells of renal tubules and respiratory/alimentary tracts [129]. This peptide can be found in saliva, cervical mucus, breast milk, cerebral spinal fluid, tears, and secretions from nose and bronchi [129]. SLPI is a 107-amino-acid, cysteine-rich polypeptide chain that is highly basic [129]. The C-terminals of elafin and SLPI exhibit both high homology and similar protease activity [127] and have demonstrated antimicrobial as well as anti-inflammatory activities (Table 1) [129,130].

### 6.2. Herpes Simplex Virus

Elafin and its precursor trappin-2 (Tr) both exhibit anti-HSV activity. Drannik and colleagues (2013) investigated the efficacy of elafin and Tr on HSV-2 infection using a Tr-expressing, replication-deficient adenovirus vector as well as recombinant TR/elafin proteins. Cells were either infected with the adenovirus vector or treated with the recombinant proteins prior to HSV-2 infection. When endometrial and endocervical epithelial cells were pre-treated with these molecules (TR IC_50_~0.07 µg/mL, E IC_50_~0.01 µg/mL), viral titers following HSV-2 infection were significantly reduced, with the activity of Tr being more potent at inhibiting HSV-2 than elafin [126]. A decrease in viral titers correlated with a decrease in production of pro-inflammatory cytokines, whereas the antiviral IFN-β response was increased. Furthermore, the recombinant molecules decreased viral attachment to cells [126]. In vivo, Tr-transgenic mice, mice generated to express the full elafin/Tr human gene, demonstrated reduced central nervous system viral load and TNF-α expression upon intravaginal infection [126]. The data provides evidence for elafin and its precursor’s potential as anti-HSV therapeutics.

### 6.3. HIV

Both proteases, elafin and SLPI, can provide protection against HIV-1 in vitro. SLPI in saliva has demonstrated anti-HIV-1 properties in vitro [131]. Genital secretions of females that are HIV-resistant contained significantly higher levels of elafin/Tr as compared to uninfected females [132,133,134]. In addition, elafin/Tr is found in and produced by epithelial cells of uterine, fallopian tubes, and cervix [132]. Ghosh et al. (2010) [132] investigated the ability of elafin/Tr in the reproductive tract to inhibit HIV-1and demonstrated HIV-1 inhibition was dose-dependent, particularly when HIV-1 was pre-incubated with elafin/Tr (10 ng/mL), suggesting the HIV-1 inhibition is mediated by direct interaction of elafin/Tr with the virus [132]. However, a recent report on multiple groups of HIV-infected women found no correlation between anti-HIV activity of mediators such as elafin and HIV susceptibility [135].

## 7. Hepcidin

### 7.1. Expression

Hepcidin, also known as human liver expressed antimicrobial peptide-1 (LEAP-1), is a 25-amino acids long AMP that is predominately expressed by liver hepatocytes [136]. It was first isolated in human blood filtrates. Hepcidin is synthesized as a pre-propeptide with two cleavage sites. The first cleavage site releases an N-terminal endoplasmic reticulum signal sequence while the second cleavage site releases the mature hepcidin peptide from a prodomain [136]. Hepcidin plays a major role in iron regulation and in systemic iron homeostasis in hepatocytes and other cells [136,137,138]. Hepcidin regulates iron uptake and efflux by directly binding to the iron exporter ferroportin, resulting in degradation of this transporter [137]. Consequently, high levels of hepcidin result in inhibition of iron uptake and sequestration of blood iron in macrophages, whereas low levels of hepcidin result in excessive uptake and toxic accumulation of dietary iron [137,138]. Hence, increased serum iron levels increase hepcidin expression. The vital role of hepcidin in iron homeostasis can determine the outcome of infections by limiting iron availability to invading pathogens.

Relatively little information is available on the effects of hepcidin on the pathogenesis of viral infections; however, hepcidin induction has been reported following a number of human and murine viral infections [137,138]. Hepcidin mRNA levels were significantly increased in mice during influenza virus PR8 infection [139]. This induction appeared to be IL-6 dependent as IL-6 knockout mice did not induce the expression of hepcidin mRNA. In addition, in primary hepatocytes that were stimulated with pathogen-associated molecular patterns (PAMPs), hepcidin was inhibited by the addition of IL-6 neutralizing antibodies [139]. These results indicate that hepcidin expression is increased following viral infections and infection-induced inflammatory responses, particularly IL-6 due to the central role this cytokine plays in hepcidin production.

### 7.2. Antiviral Activity

Liver injury as well as chronic liver infections, such as hepatitis B and C, can result in abnormal hepcidin expression. Hepcidin expression was found to be highly elevated in chronic hepatitis B and C patients, as reported by Wang and colleagues (2013). In patients with increased HBV DNA, hepcidin and IL-6 levels were elevated; however, an exact correlation between hepcidin and IL-6 was not determined [140]. In contrast, in studies assessing hepcidin levels in HBV and HCV acute infections during primary viremic phases, hepcidin levels were not upregulated and hypoferremia was not evident [141]. This difference could be attributed to the different stages of disease where hepcidin may be induced at later stages of infection when iron levels are elevated. In the same study Armitage et al. also measured hepcidin levels in HIV-1 positive plasma donors of both acute infections and patients transitioning to chronic infections. In the former group, hepcidin levels were increased and peaked with peak viral load and iron levels decreased due to retention in cells by hepcidin activity; whereas in the latter group, hepcidin levels remained elevated even in individuals undergoing ART [141]. The increase in hepcidin levels coincided with an increase in inflammatory cytokine expression [141]. HIV replication is iron-dependent and the hepcidin-induced sequestering of iron in cells such as lymphocytes and macrophages is a highly favorable condition for HIV pathogenesis.

## 8. Antimicrobial Neuropeptides

### 8.1. Expression

Neuropeptides (NPs) are low molecular weight, cationic, amphipathic AMPs. Traditionally NPs are thought to transmit and modulate signals in central and peripheral nervous systems. However, in recent years, NPs have demonstrated antimicrobial activities [142,143]. The NP, α-melanocyte-stimulating hormone (α-MSH) exhibits both antiviral and anti-inflammatory activity (Table 1). α-MSH is a 13-amino acid peptide that is a product of posttranslational processing of the precursor molecule pro-opiomelanocortin (POMC) [144]. α-MSH is widely expressed in peripheral brain tissue and by numerous cells including monocytes, dendritic cells, melanocytes, pituitary cells as well as T lymphocytes [142,144]. The active component of α-MSH resides in the C-terminal tripeptide (11-13, KPV) [144].

### 8.2. HIV

In HIV-infected monocytes, α-MSH exhibited inhibitory properties against HIV-1. Previously, α-MSH inhibited pro-inflammatory cytokine production such as TNFα and IL-1β in blood from HIV-infected individuals [145]. Barcellini et al. [55] aimed to determine if α-MSH exhibits anti-HIV properties by utilizing chronically infected monocytic U1 cells and acutely infected monocyte-derived macrophages. Not only did U1 cells consistently produce α-MSH, but α-MSH and the tripeptide (KPV) both (10 µM) inhibited HIV expression at low concentrations comparable to physiological concentrations of α-MSH, with inhibition more pronounced at higher concentrations [146]. In addition, both peptides inhibited nuclear factor-kB (NF-kB) activation which is known to enhance HIV viral protein expression.

## 9. Therapeutic Potential and Challenges of AMPs in Clinical Applications

In recent years the antiviral properties of AMPs, both naturally occurring and synthetic, and their therapeutic potential have generated increasing interest within the academic and pharmaceutical communities. Many of the AMPs originally thought to demonstrate only antibacterial activity have been found to exhibit antiviral and immune modulatory capabilities. While to date few AMP-based therapies have been clinically approved, so far, the suitability and efficacy of only a few AMPs as therapeutic agents has been studied in clinical trials as treatments for infections as well as agents for immune modulations [147]. Clinical studies utilizing AMPs include defensin-mimetic compounds (CTIX 1278) for the treatment of drug-resistant *Klebsiella pneumoniae*, an alpha-helical AMP (PL-5) for gram negative and positive bacterial skin infections, and a cationic peptide (DPK 060) for skin dermatitis [148]. AMPs are promising therapeutics due to their relatively low toxicity and tolerance in vivo. AMPs can be delivered in a variety of ways including intravenous injections, oral administration, as topical ointments, and via inhalation. In respiratory tract infections, supplementation and treatment with AMPs such as defensins and LL-37 may provide protection in the lungs. For example, nebulizing mice with LL-37 prior to infection with IAV can reduce disease severity and result in increased survival rates [50]. The same concept could be applied against other respiratory pathogens such RSV and rhinovirus with the inclusion of AMPs such as lactoferrin and EDN, which have both previously demonstrated efficacy against RSV [79,121].

AMPs can also be applied as ointments, gels, or creams. For instance, a gel for the treatment of vaginal candidiasis currently in clinical trials is derived from human α-MSH [147]. As α-MSH has previously demonstrated antiviral activity against HIV [146], this form of treatment could be developed into a cheap and effective strategy to reduce HIV transmission. The treatment can be expanded to other sexually transmitted diseases, such as HSV, and include many of the other classes of AMPs demonstrating antiviral activity against those particular viruses. Equally, oral formulations or intravenous injections are more feasible for viral infections affecting internal organs. Lactoferrin has been used as an oral supplement to antiviral therapy against HCV infections and greatly enhances the effectiveness of treatment [98]. While most studies utilizing oral LF against other pathogens alone have not provided promising results, different AMPs may prove more effective against different pathogens. An intravenous treatment of human LF is currently in clinical trials for the treatment of bacteremia and fungal infections [147]. Interestingly, the protease inhibitor Telaprevir, which is marketed for use against HCV suggests that eosinophil peptides that have dual functionality as protease inhibitors and AMPs may possess anti-HCV properties. In addition, AMPs that directly target viruses can serve as alternatives to antivirals for which resistance has become an issue as it is a challenge for viruses to change their envelope lipid compositions. Nonetheless, more research and investigation are required to assess the full potential of AMPs as novel therapeutics.

However, despite their appeal and several successful in vitro outcomes following the use of AMPs as antiviral therapeutics, their greater application as therapeutics faces many hurdles. Little clinical data on the use, toxicity, efficacy, methods of delivery, or host uptake and the in vivo response to AMPs is currently available to guide the development of AMPs into therapeutic products [147,149]. It is unclear whether and to what extent laboratory findings will translate into antiviral activity in vivo. Additionally, the relationship between peptide concentrations used in vitro experiments and physiologically effective concentrations required for in vivo activity is unknown for many AMPs. As an example, the anti-bacterial activities of AMPs appear to heavily depend on the environmental conditions used during experiments [147]. Several AMPs have been reported to control group A Streptococcus at physiological concentrations which are ineffective in vitro [150], suggested to be partly due to the physiological ionic environment not being recapitulated fully in laboratory experiments [151]. Moreover, AMPs minimally active in in vitro assays have been shown to efficiently control infections upon in vivo administration [152,153], highlighting a lack of clear correlation between efficacy and dose-dependence of AMPs derived from laboratory experiments and animal models of infection. Further research into the correlation between antiviral activity of AMPs in vitro and the translation of this activity into in vivo efficacy will be needed for further development of AMPs as a new class of therapeutics.

Another important challenge is isolation and large scale manufacturing of AMPs. The high cost of producing AMPs impedes the applicability for clinical use as a commercial-scale manufacturing platform does not yet exists. The complex secondary structures of AMPs also serve as an obstacle for large-scale production as their activity is structure-based. The use of expression systems such as bacterial systems can generate “correctly folded peptides” in large quantities; however, the peptides are susceptible to proteolytic degradation in these systems [17]. To turn AMPs into viable therapeutics for clinical use, these production disadvantages must be overcome. Meanwhile, as an alternative, non-human AMPs are being used. For example, numerous studies are examining the activity of LF utilize bovine instead of human LF due to ease of producing bovine LF in bulk. While bovine LF demonstrates equivalent and at times enhanced efficacy, as compared to the human form, other AMPs may not function similarly or may contain sequence variations that limit or abolish their properties. Synthetic peptides may prove to be a practical alternative; however, they are expensive to generate, and future use in therapies will require development of novel synthesis methods [154,155]. Several systems have demonstrated promise in mass-production of AMPs including mammalian cells and transgenic plants. While mammalian cell culture systems are preferred to produce human AMPs, they are not very cost-effective. Plant bioreactors; however, are a cost-effective method for commercial-scale AMPs production that have demonstrated applicability with other compounds [17]. Another interesting approach that could possibly counteract these challenges that has recently gained attention is the employment of agents to induce or increase expression of AMPs. For example, vitamin D induces LL-37 production that can be used to boost the natural response to infection and has been suggested for use prior to potential DENV outbreaks [43].

Furthermore, the metabolic stability of AMPs may limit their future clinical application [156,157,158]. There is a lack of data assessing the pharmacokinetics of AMPs to address issues such as the physiological half-life of peptides, the necessary dosing regiments, and the aggregation of peptides in vivo. For example, AMPs in oral and topical formulations will be subjected to enzymatic degradation, changes in pH and ionic concentrations, and face hurdles upon delivery, such as penetration of and uptake from the skin, intestinal, or nasal mucosa [159]. Intravenous administered AMPs will also face proteolytic degradation from enzymes in plasma and tissues, although the relatively shorter half-life of AMPs when compared to small-molecule drugs may be considered beneficial in some contexts due to lack of accumulation in tissues [159]. In addition, some AMPs are sensitive to salt or serum, a factor that substantially reduces their activity further limiting their applicability in clinical use.

The delivery of sufficient amounts of AMPs to an infection site may also present challenges and require careful calibration of dosing. For example, topical application of AMPs to the skin may require large-scale application of ointments, raising issues about sensitization and development of allergies against applied AMPs. Similarly, AMP activity in the context of natural innate immunity occurs in the presence numerous feedback pathways that collectively calibrate the anti-pathogen response to an appropriate level. It remains to be determined how the introduction of potentially high doses of AMPs during infections will interact with these pathways and modulate the activity of AMP therapeutics, especially as synthetic AMPs appear to elicit weaker immune responses upon administration when compared to the response produced by naturally secreted peptides during infection [160].

While these challenges may considerably impede clinical development of AMPs, the incorporation of innovative formulations can speed the process of bringing AMP products to clinical phases. The use of nanoparticles (NPs) as delivery vehicles may greatly improve the metabolic stability of AMPs. Nanoparticles can provide unique advantages, such as protection of AMPs from proteolytic degradation and control over the rate of peptide release. Several nanoparticles have been evaluated for delivery of AMPs. For example, self-assembling hyaluronic acid (HA) nanogels have been used to successfully deliver LLLKKK18, an analog of LL-37 via intra-tracheal administration in tuberculosis-infected mice [161]. Additionally, the HA nanocarrier was able to both stabilize the peptide and decrease peptide cytotoxicity in macrophages in vitro [161]. Poly lactic-co-glycolic acid (PGLA) NPs were also successfully utilized to deliver cationic AMPS [162]. Although these formulations have only been investigated in experimental animal models and in vitro applications, the outcomes for developing future AMP-based therapeutics appear promising.

## 10. Conclusions

Progress has been made in the last decade to elucidate the mechanisms of action of various AMPs. The primary mechanism of AMP-mediated antiviral activity has been attributed to direct interference with, and destabilization of, viral envelopes. However, AMPs have also demonstrated selective immune modulation. Antiviral activity against both enveloped and non-enveloped viruses has been reported with the latter hinting at the presence of undiscovered activities of AMPs, in addition to the known direct interaction with viral envelopes. Indeed, antiviral activity has also been reported at post entry steps affecting later stages in the viral life cycle, such as genome replication and viral protein trafficking. Additionally, studies have demonstrated that AMP treatment prior to viral infection results in peptide retention and internalization by cells which may reflect a more robust response to viruses compared to other potential therapeutics in development. Additionally, post-infection treatments have been reported to exhibit antiviral activity; however, to a lesser extent to that of treatments prior to infection. Nonetheless, these treatments play a role in altering viral replication and assembly as well as a role in accelerating immune activation or suppression. Hence, AMPs can directly impact viral infections or can modulate host processes that ultimately impact viral replication negatively. AMPs have been reported to drive interferon β (IFNβ) signaling, contributing to the induction of an antiviral state in susceptible cells. This dual functionality of AMPs is advantageous as they can be used as a prophylactic and/or as part of post-exposure antiviral measures. In vulnerable individuals, prophylactic expression of AMPs has the potential to become a preventative strategy against viral infections, especially during emerging pandemics. In addition, the simplicity of AMPs makes the development of synthetic peptide analogues a cost-effective measure to treat established viral infections. AMPs and their synthetic derivatives are a promising avenue to yield new strategies to control and treat a wide range of viral diseases but their application is still at the preliminary stages. Therefore, further research is warranted to understand AMP antiviral activity both in vivo and in vitro and to determine underlying mechanisms involved in AMP-mediated immune modulation for clinical applications.

## Figures and Tables

**Table 1:** Mechanisms of actions of antiviral AMPs.

AMP Family	Target	Proposed Mechanism of Action	References
Defensins	HAdV	Direct interaction with virions; reduction of cell trafficking; direct binding to cell receptor blocking entry (HS); inhibition of protein kinase C signaling; release inhibition of viral components from endosomes; decrease in proinflammatory cytokine production.	[8,10,13,16,21,22,23,24,25,26,27,28,30,31,32,33,34,35,36,37,38,39,40,41]
	HIV		
	HSV		
	RSV		
	HPV		
Cathelicidin (LL-37)	HIV	Direct interaction with virions; Increase in type I IFN expression; decrease in proinflammatory cytokine production.	[9,11,37,41,43,44,46,47,48,49,50,51,52,53,54,55,56,57,58,59,60,61,62,63,64,65,66,67,70,163]
	DENV		
	RSV		
	HRV		
	VACV		
	HSV		
	ZIKV		
	HCV		
	VEEV		
Transferrin	RSV	Direct interaction with virions; inhibition of viral attachment/absorption; delay in viral protein synthesis; Inhibition of cellular trafficking; direct binding to cell receptor blocking entry (HS and DC-SIGN).	[71,72,73,74,75,76,77,78,79,80,81,82,83,84,85,86,87,88,89,90,91,92,93,94,95,96,97,98,99,100,101,102,103,104,105,106,107,108,109,110,111,112,113,114,115,116,117,118,164]
	IAV		
	HPIV		
	HAdV		
	HSV		
	HCV		
	HBV		
	HIV		
	Hantavirus		
	HPV		
	Rotavirus		
	JEV		
	SFV		
	SINV		
	DENV		
Eosinophil proteins	RSV	Direct interaction with virions	[119,120,121,122,123,124]
	HV		
AMPS from Immune cells	HSV	Direct interaction with virions; increase in type I IFN expression	[125,126,127,128,129,130,131,132,133,134,135]
	HIV		
Hepcidin	HBV	Sequester iron from pathogens	[136,137,138,139,140,141]
	HCV		
	HIV		
Antimicrobial Neuropeptides	HIV	Inhibition of NF-kB and cytokine production	[142,143,144,145,146]
